# Supplementary figures and images for: Hippocampal pyramidal cells of the CA1 region are not a major target of the thalamic nucleus reuniens
Source: PLoS Biol. 2025 Oct 13;23(10):e3003419. doi: 10.1371/journal.pbio.3003419 (PMC12530533; doi:10.1371/journal.pbio.3003419)

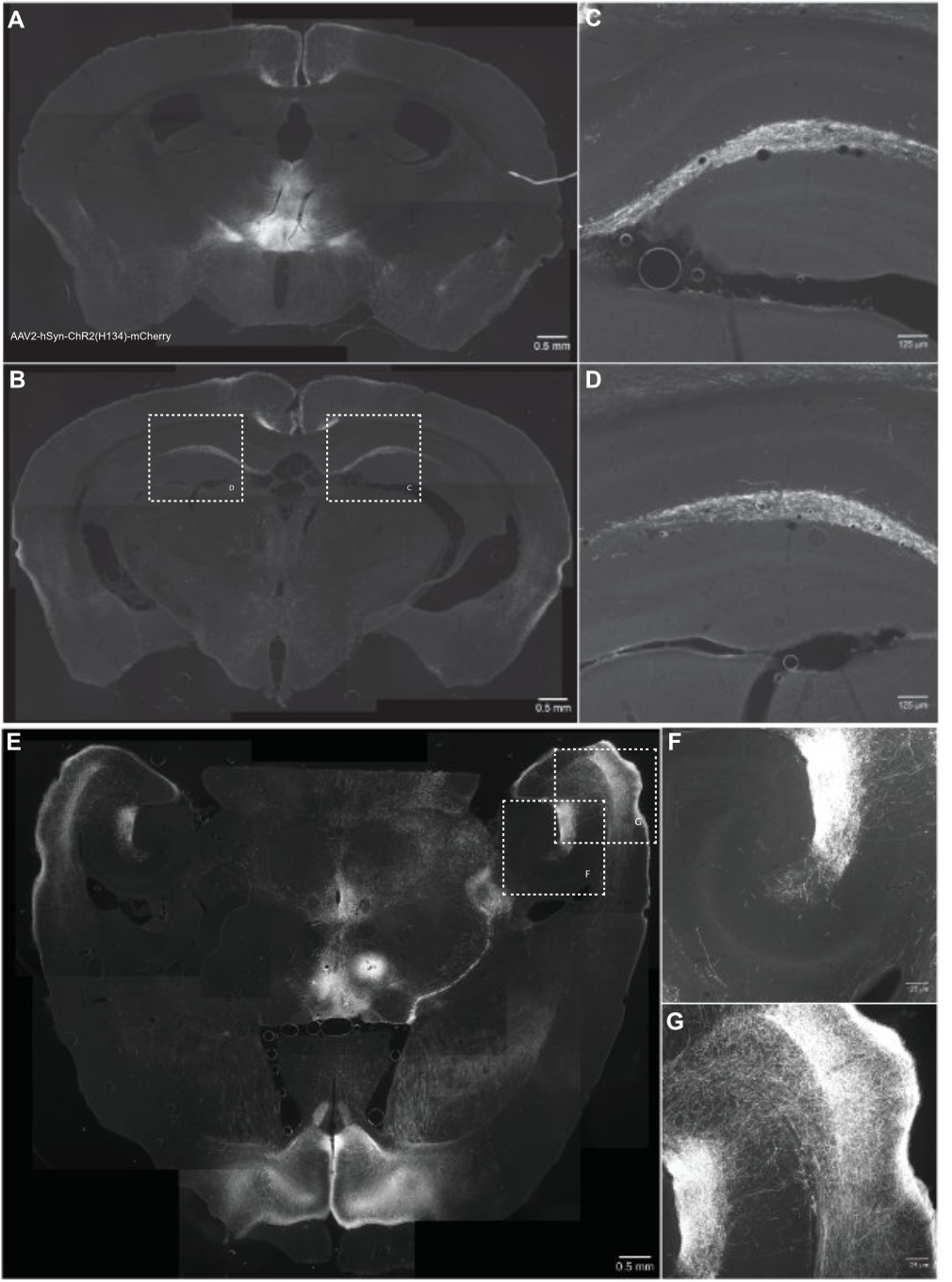

Supplement: S1 Fig — A, coronal section, showing injection site, B, more caudal section, showing the fibers in dorsal hippocampus, localized to S-LM. C and D, show hatched areas from B, on a higher magnification. E, horizontal slice, showing dense projections from nucleus reuniens in ventral prefrontal cortex, ventral CA1 and subiculum (F) and entorhinal cortex (G). (TIFF) [file pbio.3003419.s001.tiff]

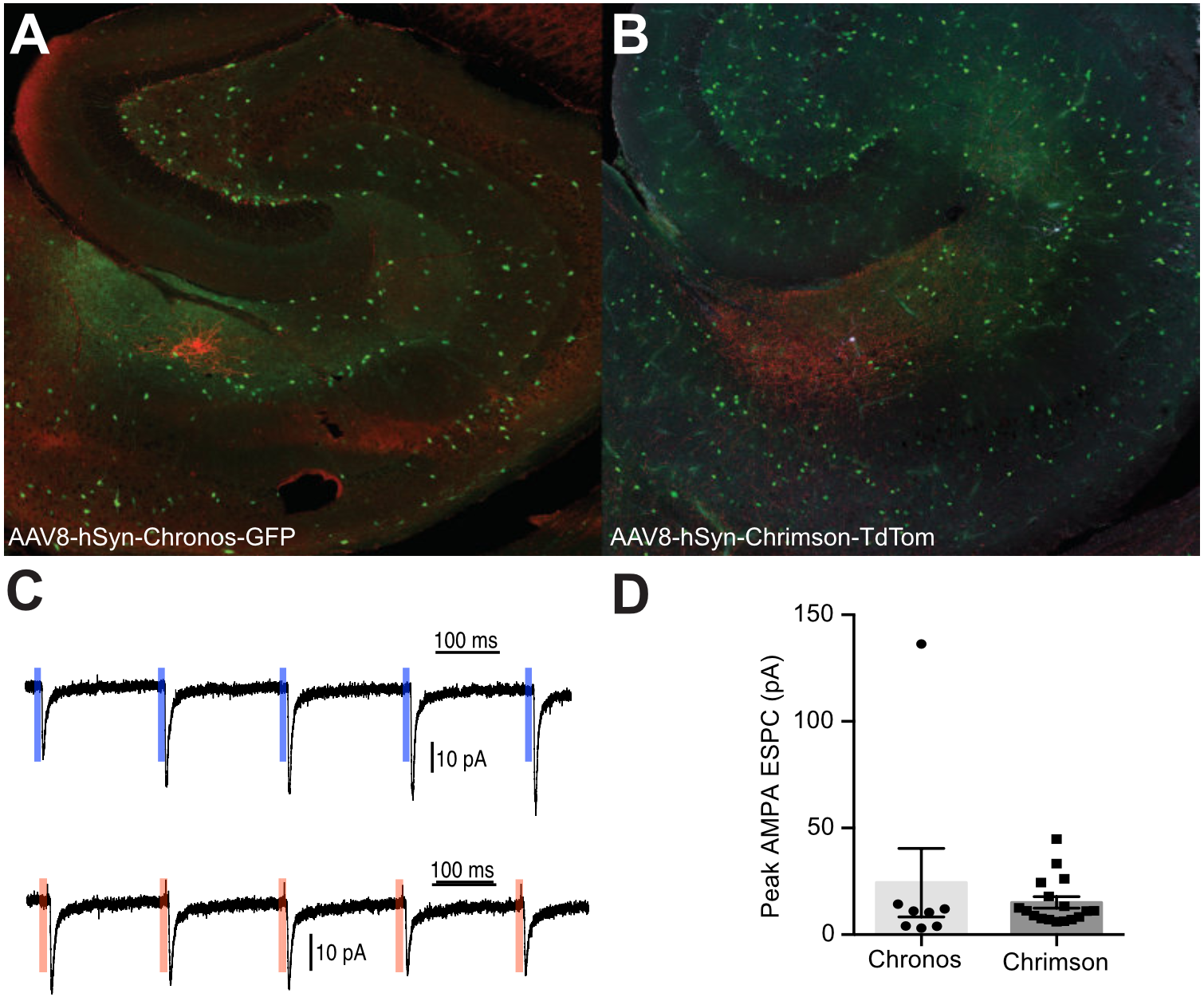

Supplement: S2 Fig — A, representative image of Chronos-GFP NRe fibers (green) in a Htr3a-GFP mouse. B, representative image of Chrimson-TdTom NRe fibers (red) in a Htr3a-GFP mouse. C, representative traces for NRe-EPSCs (AMPA-mediated) in CA1 CGE-derived neurogliaform cells from NRe axons transduced with either AAV8-hSyn-Chronos-GFP (upper trace) or AAV8-hSyn-Chrimson-TdTom (lower trace). D, no significant difference in median peak AMPA-mediated NRe-EPSC current between fibers expressing Chronos or Chrimson (Chronos versus Chrimson: 10.8 (IQR: 4.0–13.7, n = 8) versus 11.2 (IQR: 7.3–21.2, n = 17); p = 0.4143, Mann–Whitney test). These data can be found in the S1 Data. (TIFF) [file pbio.3003419.s002.tiff]

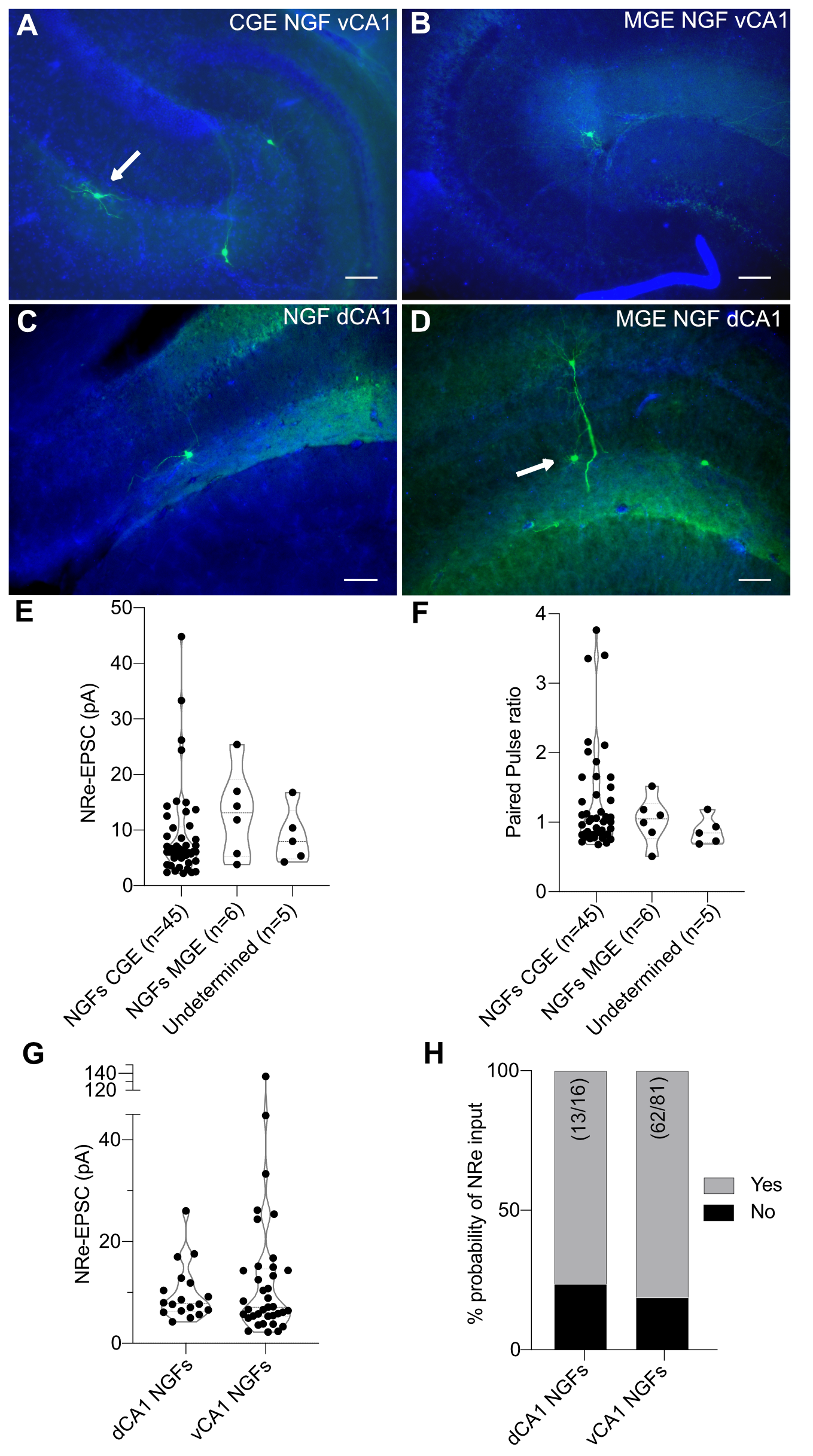

Supplement: S3 Fig — Representative images of A, CGE-derived NGF in vCA1 (arrow); B, MGE-derived NGF in vCA1; C, putative CGE-derived NGF in dCA1 (TdTom −ve in Nkx2.1-cre:Ai9 mouse); D, MGE-derived in dCA1 (arrow). E, AMPA-R mediated NRe did not vary significantly between NGFs of different embryonic origin (CGE vs. MGE vs. indeterminate origin: 9.12 ± 1.25 pA vs. 13.0 ± 3.2pA vs. 8.94 ± 2.23pA; p = 0.34, Kruskal–Wallis test). Similarly, F, NRe-EPSC paired pulse ratio did not vary by embryonic origin (CGE vs. mGE vs. indeterminate origin: 1.27 ± 0.11 vs. 1.03 ± 0.14 vs. 0.88 ± 0.09; p = 0.39, Kruskal–Wallis test). G, mean AMPA NRe-EPSC did not significantly differ between dCA1 and vCA1 NGFs, pooled across embryonic origin (dCA1 vs. vCA1, 9.88 ± 1.3 pA vs. 14.3 ± 3.7pA; p = 0.6504, Mann–Whitney test). H, probability of NRe input in neurogliaform cells in dorsal and ventral CA1 was 76.5% and 81.3%, respectively. These data can be found in the S1 Data. (TIFF) [file pbio.3003419.s003.tiff]

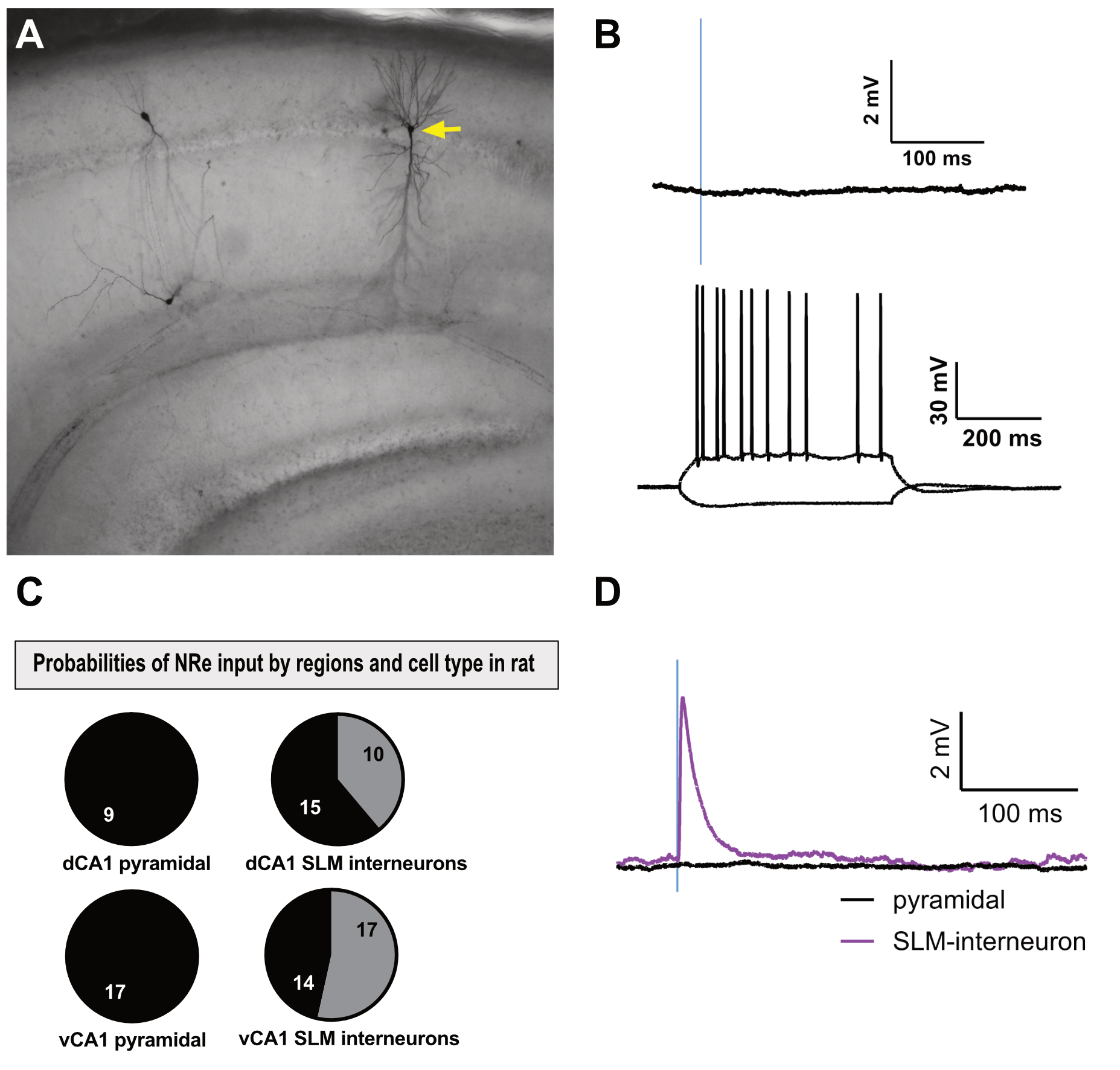

Supplement: S4 Fig — A, post hoc recovery of CA1 neurons in dorsal HPC of rat. B, lack of EPSP for pyramidal cell shown in A in response to optogenetic stimulation (top), plus response to depolarizing and hyperpolarising current steps (bottom). C, total connection probabilities for CA1 pyramidal cells and GABAergic neurons in SL-M, with positive responses in gray and null responses in black. Data from 21 rats. D, example EPSP from an SLM-interneuron (purple) and a pyramidal cell (black) recorded from vCA1. (TIFF) [file pbio.3003419.s004.tiff]

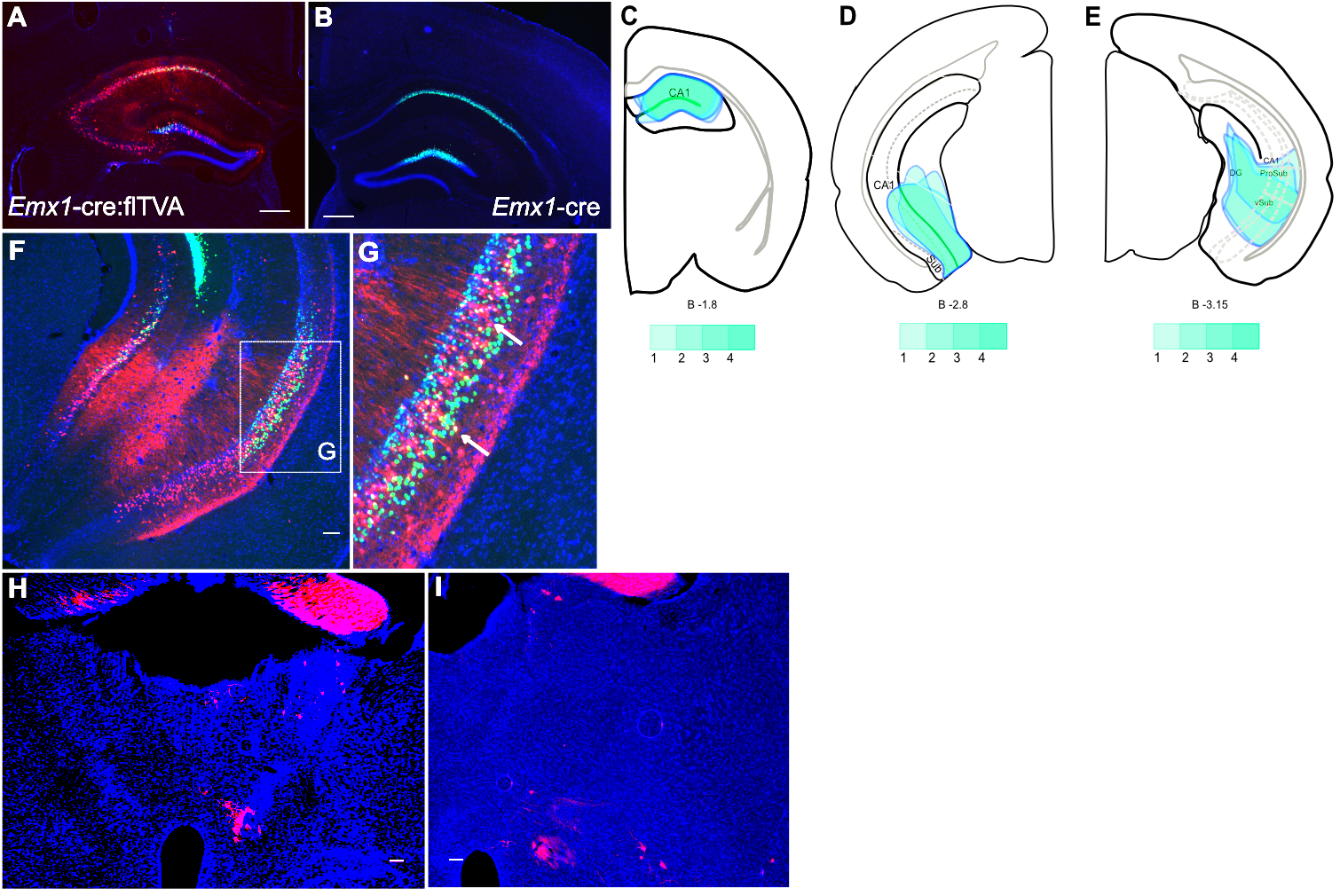

Supplement: S5 Fig — A, representative example of injection site for retrograde labeling viruses in Emx1-cre:TVA mouse. Both green (transduced by the helper AAV virus) and red (transduced by the pseudotyped rabies virus) cells can be seen. B, in a control mouse (Emx1-cre) without TVA, the pseudotyped rabies virus was unable to enter CA1 pyramidal cells (n = 6 mice). Scale bar represents 100 μm. C–E, schematic images showing spread of viral representative spreads of virus for injections in the dCA1 (C), vCA1 (D), and vSub (E) regions. Each figure is a composite of 4 injections, with the darker color indicating more mice. F and G, representative example of injection on low (F) and high (G) magnification of injections that spread between vCA1, prosubiculum, and vSub. H and I, retrogradely-labeled neurons were present in NRe only in mice with starter cells located in prosubiculum and subiculum, confirming our observations from anterograde optogenetics experiments (Fig 1). (TIFF) [file pbio.3003419.s005.tiff]

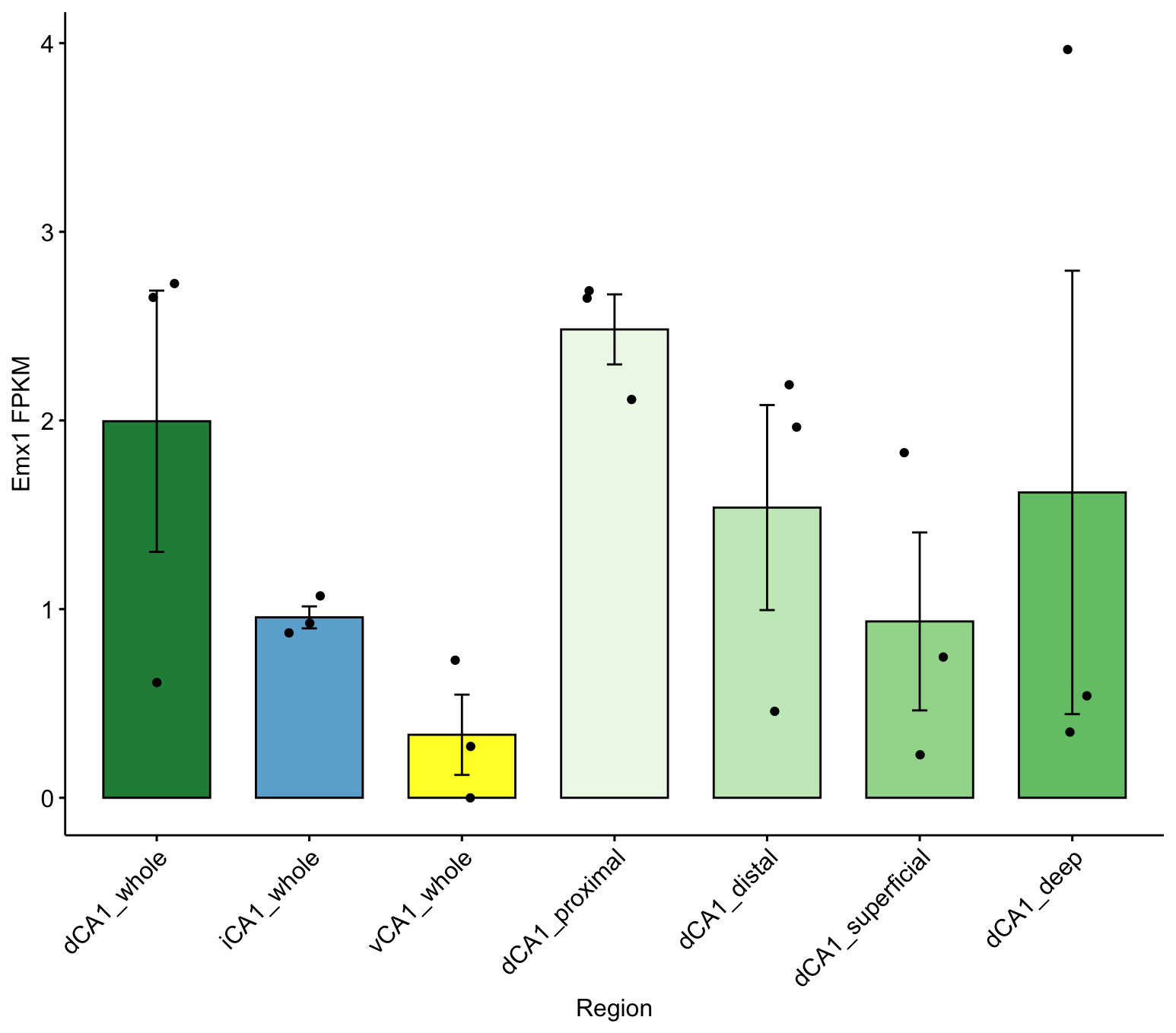

Supplement: S6 Fig — Secondary analysis of Cembrowksi and colleagues, 2016, https://doi.org/10.1016/j.neuron.2015.12.013) revealed that Emx1 expression is ubiquitous throughout HPC, albeit at (nonsignificantly) lower levels in ventral CA1 compared with dorsal or intermediate CA1. These data can be found in the S1 Data and the code generating the plots and associated analysis can be found in S1 Code. (TIFF) [file pbio.3003419.s006.tiff]
