# Supplementary material for: Hippocampal pyramidal cells of the CA1 region are not a major target of the thalamic nucleus reuniens
Source: PLoS Biol. 2025 Oct 13;23(10):e3003419. doi: 10.1371/journal.pbio.3003419 (PMC12530533; doi:10.1371/journal.pbio.3003419)
Supplement: Attachment — Submitted filename: Response to reviewers final.docx [file pbio.3003419.s011.docx]

Response to reviewers

We thank the reviewers for their insightful comments, which have helped us to further improve the manuscript. We have pasted the reviews below and included inline responses to each comment. We have highlighted where in the main text we have made the changes.

In addition, we have added an extra patch clamp electrophysiology dataset from rat hippocampus, carried out independently in the laboratory of Prof Zafar Bashir at the University of Bristol. This has replicated one of our key findings, namely the inability to evoke a nucleus reuniens-driven post-synaptic response in pyramidal cells whilst still observing responses in SL-M interneurons. While this has added a bit of time to our revision (we thank the editor and reviewers for their patience here), we feel that it significantly strengthens the findings of our work and adds a cross-species validation.

**Reviewers comments:**
 **Reviewer #1:** Review of PBIOLOGY-D-25-00109R: "Hippocampus does not appear to be a major target of thalamic nucleus reuniens" by Andrianova et al.

In this manuscript, Andrianova and colleagues examine the degree to which axons from the nucleus reuniens form synapses on neurons in the hippocampus, prefrontal cortex, and entorhinal cortex. This question is important because the RE-to-CA1 pathway is presumed to be the major pathway linking prefrontal cortical signals to the principal neurons in the hippocampus. Although some past work has found evidence for these connections, Andrianova and colleagues demonstrate that these specific connections appear absent. The authors perform these experiments using optogenetic strategies paired with slice recordings, and with data from monosynaptic rabies mapping. Collectively, the experiments are sound, the results compelling, and the interpretations important. I assume the field will appreciate the clarification about how the RE circuit nodes are connected in distinct ways to different cell types in CA1, EC, and PFC.

After review of the manuscript, I have the following recommendations/comments/concerns:

-    The title uses the terminology 'major target', but this seems hard to establish as hippocampal  neuogliaform cells (and potentially other interneuron types localized to SLM) appear to consistently receive RE input. I would favor clarifying it with 'Hippocampal pyramidal neurons do not…' (though the subiculum is arguably part of the hippocampus) or better yet by emphasizing the role of inhibition, such as 'Interneurons in CA1 are the major hippocampal target of thalamic nucleus reuniens'

*We thank the reviewer for this thoughtful and helpful suggestion. We agree that the title was perhaps a bit general. Our thinking was that even in pyramidal cells where we could evoke a response from reuniens (i.e. prosubiculum and subiculum), the magnitude of the EPSC was substantially lower than that seen in EC and PFC. But as the focus of the manuscript is hippocampus, we have amended the title in a manner similar to that suggested by the reviewer to capture the key essence of the findings in hippocampus.*

-    The experiments by which the authors confirm the lack of input (with interleaved interneuron recordings from the same slices) provides strong evidence that the inputs are at best very sparse and otherwise absent. They should be commended for this approach. The authors discuss previous papers that suggest the presence of these inputs with proper context and treat them quite fairly. As one of those authors, I appreciate that.

*Thank you. The previous assumptions about reuniens connectivity were entirely sensible given the anatomy, and it took us quite a while to convince ourselves that the projection to CA1 pyramidal cells was indeed sparse or absent!*

-    The high probability of activating neurogliaform cells is an important mechanism by which RE might control CA1. These interneurons drive GABA-B mediated inhibition of the distal dendrites, which presumably exerts control over CA1 neurons switching from linear to non-linear forms of synaptic integration. There is some text devoted to this in the discussion, but a more thorough examination of how RE might contribute to information processing in CA1 could improve the manuscript (see more below).

*We agree that the selectivity of reuniens for CA1 neurogliaform is an exciting observation and has important implications for circuit function. We previously discussed this at length in an opinion article (*[*DOI: 10.1126/science.add2681*](https://doi.org/10.1126/science.add2681)*) and were being catious not to self-plagiarise. But we agree that the discussion in the present study would benefit from a wider discussion of the role of neurogliaform cells in NRe-dependent behaviours and have now expanded this part of our discussion.*

-    The rabies results need more methodological unpacking/description in the text, and perhaps a schematic to demonstrate how the experiments were performed (injection sites, viruses, etc.) and which colors correspond to which cells (containing helper-virus, double labeled starter cells, and connected cells). As it stands now, it takes a lot of work for the reader to understand the experiments without these details (perhaps move the C-E from Supp Fig 4 to main?). The images are very small and hard to parse- part of this is the wish to show the whole field, and part is image quality.

*We have revised and improved the images and figures from the rabies experiment, and have better described our colouring, labelling, etc.*

-    If the rabies results are already published, can the authors reproduce the data in the figures (with the text clearly stating this) to allow the reader to gauge the efficiency of the rabies labeling? Specifically, it would be nice to know the number of starter cells in CA1 relative to monosynaptic rabies labeled cells in CA3 and EC. If there are 10 starters cells and 10 EC cells, then the reader would know the labeling efficiency is very low and be able to place the lack of RE cells in context. Is this the SAD strain of psuedotyped rabies or the CVS strain? These two are very different in terms of transcellular efficiency. The authors should also discuss the possibility that rabies virus particles may not retrogradely label different axons with the same efficiency- axonal tropism for modified rabies is a relatively unexplored issue.

*We thank the reviewer for raising this point. We have now reproduced the starter cell quantification from our eLife study and have added further discussion on virus tropism, along with a better description of the viral strain used.*

-    In the concluding paragraph, I think describing the RE input patterns (or the RE-CA1 pathway) as a 'red herring' is taking it too far. The authors have not demonstrated that this pathway is unimportant for hippocampal function; in contrast, they have identified the key cellular targets of RE in CA1. Without devaluing the finding that CA1 pyramidal cells are not receiving RE inputs, such cellular targeting to interneurons selectively in CA1 may permit RE to differentially influence processing by reducing CA1 output yet increasing that of subiculum. I think more discussion on how RE-to-CA1 neurogliaform cells impact information processing might come across as a greater advance for the field.

*We agree that we somewhat over-stated the main finding and have adjusted the text accordingly.*

-    The presentation of the text and figures could be improved. For example, I believe the first sentence under the heading "Implications for NRe function" should read "hippocampal projections to the prefrontal cortex" rather than "Prefrontal cortical projections to the hippocampus", right? Figure 4 axis legends should read "AMPA" rather than "AMAP". Figure 4 and 5 have the same title, but are focused on two different regions. The pie charts are repeated across figures, and the figure organization could be improved such that Figure 3 is not referred to in the text before Figure 2.

*We apologise for the typos in the figures. We have tidied up the text and figures as suggested by the reviewer. The organisation of the figures (3 being referenced before 2) was unavoidable as, for clarity, we chose to present ventral and dorsal hippocampus data in separate figures (1 and 2, respectively), with summary data separately in figure 3. Various early drafts of the manuscript had all of these data in a single figure, but it was too large and unwieldy, so the presentation of data we went with in the end felt easiest on the reader.*

**Reviewer #2:**

In the current manuscript Andrianova and coauthors analyze the projections between the nucleus reuniens (NRe) and hippocampus. To this end they integrated ex vivo optogenetics and electrophysiology, alongside monosynaptic circuit-tracing. Based on the performed experiments they came to very surprising and exciting (if true) conclusion that  the prefrontal and entorhinal cortices,  subiculum and prosubiculum, but not the CA1 pyramidal neurons, receive the direct input from NRe. Trying to understand the source of discrepancy between this work and former studies which identyfied synapses between NRe and pyramidal CA1 neurons (Wouterlood et al., 1990; Herkenham, 1978; Kajiwara et al., 2008; Vertes et al., 2006) I came to conclusions that the authors should possibly more precisely define what they

studied.

1.    One of the source of the controversy can be definition of the prosubiculum and subiculum areas. For example, The Mouse Brain in Stereotaxic Coordinates by Paxinos and Franklin does not recognizes prosubiculum. It is just CA1 area. Clear introduction of  the prosibiculum area and a precise graphic illustration how this area was defined by the authors (both in the coronal and sagittal planes) in each experiment would be useful. For example, the areas labeled by the authors as vSub and ProSub (supplementary Fig. 4E) are named as CA1 area by Paxinos and Franklin (Figure 60, Bregma -3.52 mm), although Sub (S) is distinguished in this atlas.

- *While Paxinos and Franklin does not show prosubiculum, this region does clearly exist. We used the Lorente de Nó boundaries on the recommendation of John Aggleton, a collaborator and world-leading expert in hippocampal anatomy. We have added more description of how we defined prosubiculum, added schematic images to figures 1 and 2 to show the boundaries we used, and added a citation to Ding (2013;* [*10.1002/cne.23416*](https://doi.org/10.1002/cne.23416)*) that shows boundaries of prosubiculum across species (primate, human, mouse) that correspond closely to those used in our study. While some do find the existence of prosubiculum to be contentious, recent work as shown that it is indeed transcriptionally-distinct from CA1 or subiculum (*[*10.1016/j.celrep.2020.107648 External Link*](https://doi.org/10.1016/j.celrep.2020.107648)*; cited in our original submission). While the boundary between ventral CA1 and subiculum is ambiguous in the coronal plane, it is much clearer in horizontal sections, which is the plane in which we carried out electrophysiological recordings. We only ever saw pyramidal cells respond to reuniens axon activation when we were recording outside of the nicely ordered cell layers found in CA1. We have added a post hoc recovery image to Figure 6 (panel M), where the boundaries can be clearly seen in the horizontal section. You can observe a gradual thinning of the SR layer in prosubiculum (SR and SLM in CA1 are similar thickness), thickening of the SP layer and a start of the reduction in density and order of the pyramidal cells. The boundary between prosubiculum and subiculum is denoted by the disappearance of SR layer and transition of the dense pyramidal cell packing in SP of CA1 into a more subicular-like pattern of pyramidal cell distribution*

2.    The authors should precisely describe what parts of dCA1 and vCA1 were probed with patch clamp analysis. Judging from the pictures included in the manuscript, I am not sure if they analyzed more anterior parts of dCA1 (Bregma > -2.3 mm). This is an important issue firstly because dCA1 neurons differ functionally in the anterior-posterior axis and secondly because a recent study  (<https://eur03.safelinks.protection.outlook.com/?url=https%3A%2F%2Fdoi.org%2F10.7554%2FeLife.101736.3&data=05%7C02%7CMick.Craig%40glasgow.ac.uk%7C15fa383ecfcb4931d1d408dd5a69e274%7C6e725c29763a4f5081f22e254f0133c8%7C1%7C0%7C638766133012830591%7CUnknown%7CTWFpbGZsb3d8eyJFbXB0eU1hcGkiOnRydWUsIlYiOiIwLjAuMDAwMCIsIlAiOiJXaW4zMiIsIkFOIjoiTWFpbCIsIldUIjoyfQ%3D%3D%7C0%7C%7C%7C&sdata=Z1UC4IKCksdvYwcgX%2BNTIlWSiCT2dh%2F9fKPVg%2FF0SIc%3D&reserved=0>) showed NRe axons in close proximity to dCA1 excitatory synapses of the pyramidal neurons. However, in this work the authors focused on the anterior dCA1 (AP, Bregma from -1.7 to 2.06). This discrepancy with current data should be discussed.

*For electrophysiological experiments slices of 400 microns were used, with the first coronal slice roughly corresponding to Bregma -1.5-1.9, and we recorded from slices extending to approximately bregma -2.5, after which dorsal CA1 transitions into intermediate CA1.*

*We also thank the reviewer for highlighting the study by Ziółkowska and colleagues, which was published after we submitted the present manuscript. This manuscript does indeed show close proximity of NRe axons to PSD95-expressing dendritic spines in SL-M, which would be reasonable to assume are arising from CA1 pyramdal cells. However, Peer Wulff’s group recently (2019) described a PNN-negative population of spiny PV interneurons in CA1 that extend dendrites into SL-M. Additionally, while PSD95 is typically taken to be a marker of glutamatergic neurons, it is also found on GABAergic neurons, particularly those expressing PV, in work published by ourselves (Pelkey et al, 2015) and others. We have extended our discussion in the main manuscript to include the Ziółkowska manuscript and provided additional context and interpretation.*

3.    The information on number, age and sex of the animals in different experiments is missing. The authors just indicate that the age of animals was between 2 (when the hippocampus may be not fully developed) and 7 months.

*The age of animals used was clarified in the methods section – the minimal age of animals* ***on the day of the stereotaxic injection*** *was 2-5 months old, meaning the earliest age of animals from which ex vivo slices were made for patch clamping was 3 months old. Whilst 2 months is a widely accepted young adult time point for a mouse, we hope that this clarification would help. The details of animals’ age/sex and numbers have now been added to the methods section. We apologise for this oversight.*

Minor issues:
Title: The authors would like to conclude that Hippocampus does not appear to be a major target of thalamic nucleus reuniens. However, based on theirs current and published data neurogliaform cells in the hippocampus are the major target of NRe.
Fig 2. (A) Please, mark boundaries between prosubiculum, subiculum and Ca1.
(C) and (F) Please, label the neurons on the microphotographs. Especially, on the microphotograph F it is not clear which pyramidal neuron is in CA1 and which in prosubiculum.

*Boundaries on Figure 2 have been added and all neurons were labelled.*

Fig. 3. Please, clarify what is n value - cell or animal?
(C) This is unclear why the authors use 2-way ANOVA to analyze data demonstrated on this graph. What are two factors possibly contributing  to AMPA currents here?

*In all cases N=cells, a note was added to each relevant figure legend. Figure 3C – the typo was corrected – it was indeed a one-way ANOVA test that was used for comparisons in this instance.*

Fig. 4. Data shown in panel A.iv seem to a replicate of what is shown in Fig. 3A.
The information how many animals were tested, their age and sex is missing.
Fig. 6. The punctuation marks in the legend are confusing. As opposite to earlier legends, here the names of panels (letters) are after the pane description. The text has commas instead of periods.
(J) Please, show microphotographs with higher magnification so the morphology of NRe neurons could be clearly seen.

*Figure legends for Figures 4 have been corrected, duplicate pie chart data removed. Information on number, age and sex of animals used for each dataset was added to the methods (slice preparation and electrophysiology subsection). Figure legend 6 formatting has been altered to match the formatting of the other figure legends. An inset magnified image of the cells in NRe is added to panel L (previously panel J).*

Materials and methods. 
-    Catalog numbers of used chemical are missing. 
-    I believe that the authors used  Nikkon Eclipse E800, not Nikkon 800 microscope. Please, confirm and correct. Please, indicate also what objectives were used.
The font at the scale bars in supplementary figures 1 is too small to read and the scale bars are too slim.

*All catalog numbers for chemicals have been added and details for the microscope and objectives used added to the methods section. Font size and scale bars on Supplementary Figure 1 have been increased.*
